# Hidden Benefits of Electric Vehicles for Addressing Climate Change

**DOI:** 10.1038/srep09213

**Published:** 2015-03-19

**Authors:** Canbing Li, Yijia Cao, Mi Zhang, Jianhui Wang, Jianguo Liu, Haiqing Shi, Yinghui Geng

**Affiliations:** 1 College of Electrical and Information Engineering, Hunan University, Changsha, 410082 China; 2 Centre for Systems Integration and Sustainability, Michigan State University, RM 115, S. Harrison RD, East Lansing, MI 48823 USA; 3 Centre for Energy, Environmental and Economic Systems Analysis, Argonne National Laboratory, 9700 S. Cass Avenue, Bldg. 221, Argonne, IL 60439 USA

**Keywords:** Climate sciences, Environmental sciences

## Abstract

There is an increasingly hot debate on whether the replacement of conventional vehicles (CVs) by electric vehicles (EVs) should be delayed or accelerated since EVs require higher cost and cause more pollution than CVs in the manufacturing process. Here we reveal two hidden benefits of EVs for addressing climate change to support the imperative acceleration of replacing CVs with EVs. As EVs emit much less heat than CVs within the same mileage, the replacement can mitigate urban heat island effect (UHIE) to reduce the energy consumption of air conditioners, benefitting local and global climates. To demonstrate these effects brought by the replacement of CVs by EVs, we take Beijing, China, as an example. EVs emit only 19.8% of the total heat emitted by CVs per mile. The replacement of CVs by EVs in 2012 could have mitigated the summer heat island intensity (HII) by about 0.94°C, reduced the amount of electricity consumed daily by air conditioners in buildings by 14.44 million kilowatt-hours (kWh) and reduced daily CO_2_ emissions by 10,686 tonnes.

## Introduction

The replacement of CVs by EVs has been an inevitable trend around the world. As of December 2013, there were 405,000 highway-capable plug-in electric passenger cars and utility vans worldwide^1^. An increasingly hot debate on whether the replacement of CVs by EVs should be delayed or accelerated has surfaced among researchers, enterprises and governments^2^, since EVs are more costly and cause more pollution than CVs in the manufacturing process^3,4^.

UHIE is influential in metropolitan areas^5^. For example, the surface temperatures in some urban areas of Beijing, on July 5, 2010, were nearly 50°C^6,7^. UHIE, which contributes to the extremely high temperatures in urban areas, is the main cause of this phenomenon.

UHIE would cause huge air-conditioning energy consumption^8,9,10^. The positive feedback of air-conditioning energy consumption to UHIE was proposed and evaluated in Refs. 11, 12, 13. Heat emitted by vehicles and air conditioners in buildings, the main source of anthropogenic heat emissions in urban areas, is one of the main causes of UHIE^14^. The strength of UHIE is measured in terms of HII^15^. HII is calculated as the urban temperature minus the rural temperature, which depends on heat emissions, aerosol pollution, underlying ground surface and ventilation, etc.

**Figure 1:**
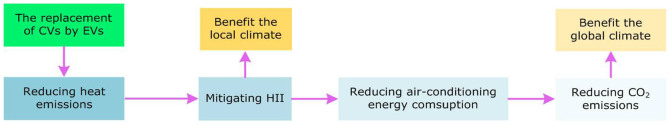
The two hidden climate benefits of replacing CVs with EVs. EVs emit much less heat than CVs within the same mileage. Replacing CVs with EVs would mitigate HII and CO_2_ emissions to benefit local and global climates.

The replacement of CVs by EVs has important implications for UHIE. There is no doubt that CVs will be replaced by EVs in the long run because fossil energy is non-renewable. However, there is an increasingly hot debate on whether the replacement should be delayed or accelerated^2^. Here we reveal two hidden benefits of EVs for addressing climate change to support the acceleration of the replacement. EVs emit much less heat than CVs within the same mileage. Therefore, the replacement can mitigate HII, which can reduce the amount of electricity consumed daily by air conditioners, benefitting the local and global climate. These effects are shown in Fig. 1 and Beijing in the summer of 2012 is taken as an example.

## Results

### Heat emissions ratio of EVs to CVs

In Beijing in 2012, the average heat emissions by CV and EV per mile were estimated to be 6.31 and 1.25 million joules (J) respectively. Then the average heat emitted by EV per mile was about 19.8% of that by CV.

### Reduction of heat emissions

In the summer of 2012 in Beijing, the daily heat emitted by CVs was 9.85 × 10^14^ J. If CVs were replaced by EVs, the heat emitted by EVs would be reduced by 7.90 × 10^14^ J and the heat emitted by power plants would be increased by 6.09 × 10^13^ J, so the total daily reduction of heat emissions would be 7.29 × 10^14^ J.

### HII mitigation and reduction of air-conditioning energy consumption and CO_2_ emissions

**Figure 2:**
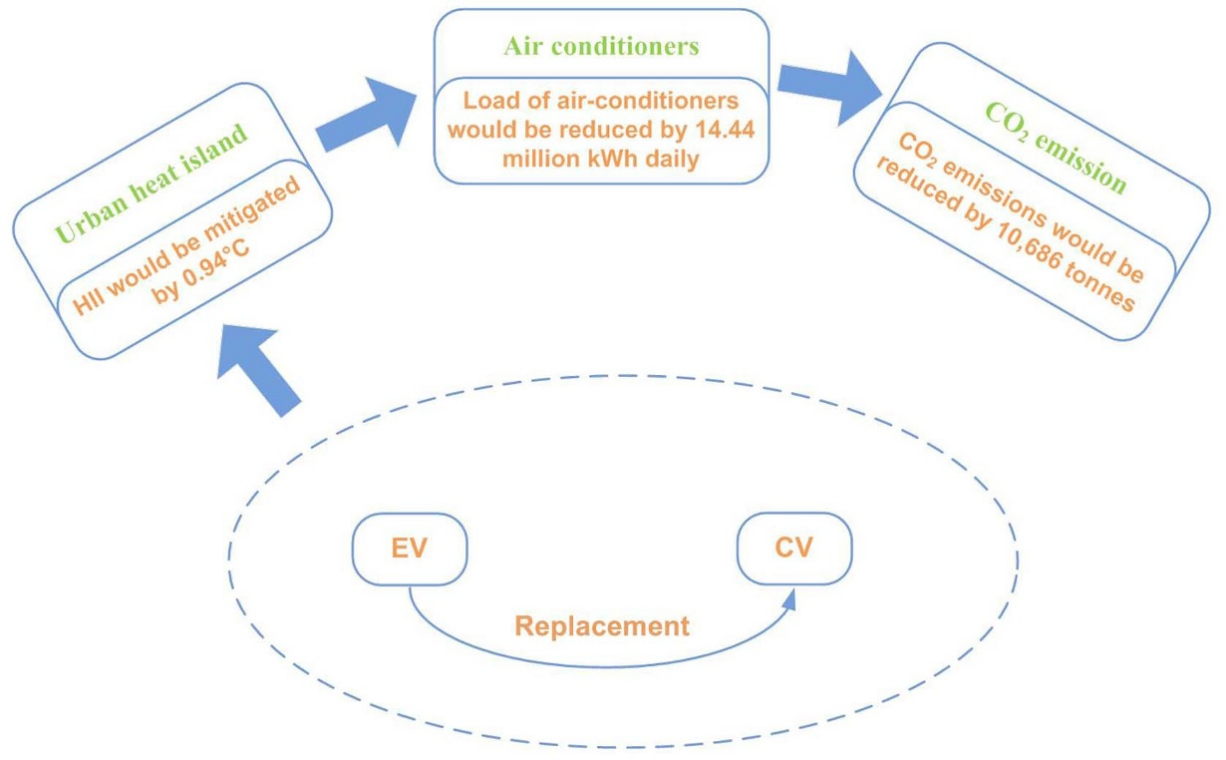
Overview of the benefits of replacing CVs with EVs. HII would be mitigated by 0.94°C, 14.44 million kWh electricity would be saved daily in summer and about 10,686 tonnes of CO_2_ emissions would be eliminated.

The average HII was estimated at 3.0°C in the summer of 2012 in Beijing. Heat emissions, which are mainly caused by vehicles and air conditioners in buildings, contributed about half of the HII in Beijing^16^. The daily heat emitted by air conditioners was 4.32 × 10^14^ J. The decreased heat emissions from the replacement are 1.69 times higher than the emissions of air conditioners in buildings, which would mitigate the summer HII by about 0.94°C (Fig. 2). Because of the reduction of HII, the energy consumed by air conditioners in buildings would decrease by 12.03%. The amount of daily energy that could be saved is 14.44 million kWh, which could reduce CO_2_ emissions by 10,686 tonnes per day (Fig. 2). The results are described in Fig. 2.

## Discussion

Air conditioners used in vehicles are dispersed and the energy consumed by them is difficult to calculate. The energy saving and CO_2_ emissions reduction are underestimated, but the benefits are still very remarkable.

According to the definition of specific heat capacity, when specific heat capacity is a constant, temperature variation is proportional to the heat variation. According to Ref. 17, at standard atmospheric pressure, the specific heat of dry air is 1.005 kJ/(kg × °C) at temperatures ranging from 0°C to 60°C. The average temperature in summer of Beijing is about 24.6°C^18^, so the specific heat capacity of air could almost be regard as a constant in our model. Thus, it is reasonable to assume that the relationship between heat emissions and HII is linear.

There are many reasons for UHIE, three of which are identified as critical factors: the difference in heat emissions, more aerosol particles and different thermal properties of the ground surfaces. It has been found that pollution aerosols have a positive impact on HII in some places^19^, while some other studies have found that aerosols have a negative impact on HII^20^. The impact of aerosol particles on HII is also highly non-linear and uncertain^21^, therefore, they are not taken into consideration in this model. As to the third factor, the replacement of CVs by EVs is a virtual replacement, which does not change the ground surfaces of Beijing, the thermal properties of the ground surfaces are regarded as unchanged in our model.

## Methods

**Figure 3:**
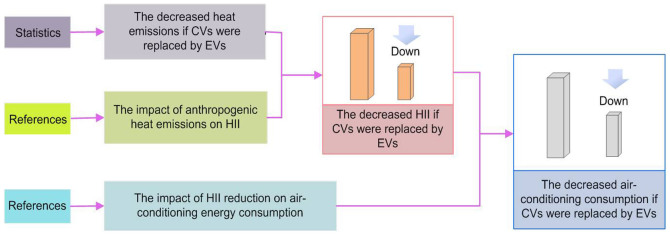
Diagram of the methods. The data source and procedure of reasoning and estimating are presented. CVs, conventional vehicles; EVs, electric vehicles; HII, heat island intensity.

The methods used in this research are summarized in Fig. 3.

First, we analysed the decreased heat emissions caused by the replacement of CVs with EVs. Second, based on the statistics of the contribution of air conditioners in buildings to UHIE and the assumed linear relationship between heat emissions and HII, we deduced the impact of anthropogenic heat emissions on HII. Finally, according to the impact of HII changes on air-conditioning consumption in buildings, we achieved the decreased air-conditioning energy consumption by the replacement.

### Heat emissions ratio of EVs to CVs

Energy consumed by CVs is all converted to heat and eventually emitted to the air. Engines of CVs convert fuel energy into thermal and mechanical energy. Then the mechanical energy is converted to heat by overcoming mechanical friction, wind and tire rolling resistance. Energy consumed by EVs is also converted to heat eventually.

In Beijing, the average fuel economy of light-duty vehicles was estimated to be 20.6 miles per gallon in 2012^12^. The heat emitted by gasoline combustion per gallon is 130 million J^22^. Therefore, the average heat emitted by CVs per mile would be:where *P*_1_ is the heat emissions per mile by a CV, *E*_1_ is the fuel economy, *Q*_1_ refers to the energy contained in a gallon of gasoline.

The electricity consumed by an EV per mile in China ranges from 18 kWh to 25 kWh per 100 kilometres for different models^23^ and the average is estimated at 0.346 kWh per mile. 1 kWh is equal to 3.6 million J. The heat emitted by an EV per mile would be:where *P*_2_ is heat emissions per mile by an EV, *E*_2_ is the electricity per mile consumed by an EV and *Q*_2_ is the energy contained in 1 kWh.

According to equations (1) and (2), heat emitted by EVs per mile is 19.8% of that by CVs, as shown in equation (3):where *r* is the ratio of heat emitted by EVs to that by CVs.

### Increment of heat emissions by power plants in Beijing

In 2012, the total electricity consumption of Beijing was 87,430 million kWh. About 28,312 million kWh was generated by thermal power plants in Beijing, accounting for 32.38% of the total electricity consumption^24^. In 2012, there were 5.2 million vehicles in Beijing^25^ and the average daily driving distances were 30 miles^23^. If CVs were replaced by EVs, the increment of electricity produced by thermal power plants (*ΔE*) in Beijing would be:where *N*_1_ is the number of vehicles in Beijing in 2012, *L* is the average daily driving miles and *e* is the ratio of electricity generated by thermal power plants in Beijing to the total electricity consumption of Beijing.

According to the statistics from Ref. 26, when 1 kWh is produced by Beijing's thermal power plants in 2012, the heat emissions would be 3.48 × 10^6^ J. Thus, if CVs were replaced by EVs, the increment of heat emissions by thermal power plants (*H*_1_) in Beijing would be:where *h*_1_ is the heat emissions from Beijing's thermal power plants when 1 kWh is produced.

### Reduction of heat emissions

In Beijing in 2012, the daily heat emitted by CVs (*H*_2_) was as following.In the summer of 2012, the average load of air conditioners in buildings was approximately 5 million kW^27^. Therefore, the daily heat emitted by air conditioners (*H*_3_) in buildings was:where *P*_5_ is the average load of air conditioners and *N*_2_ is the number of hours per day.

If CVs were replaced by EVs, the reduction of daily heat emitted by vehicles (*H*_4_) would be as following.If CVs were replaced by EVs, more electricity would be consumed. This would increase power plants' heat emissions in Beijing. Therefore, the total daily reduction of heat emissions (*H*_5_) is calculated as follows.

### HII mitigation

The average HII was 2.77°C during the summer of 2005 in Beijing^28^ and 2.90°C in 2009^29^. The data in 2012 are not available from official statistics or academic papers. According to the growth rate of HII from 2005 to 2009, we estimated HII to be 3.0°C in 2012. Heat emissions, mainly caused by vehicles and air conditioners in buildings, contributed to about half of the HII in Beijing^16^. Therefore, if CVs were replaced, in 2012 in Beijing the decreased heat emissions would reduce HII by:where ΔHII is the decreased HII resulting from the decreased heat emissions with the replacement and *k*_1_ is the contribution of heat emissions to HII in Beijing.

### Reduction of air-conditioning energy consumption

If HII were to decrease by 1°C, the energy consumed by air conditioners in buildings would decrease by 12.8% during the summer in Beijing^11^. Although the estimation in Ref. 16 is based on data from Beijing in 2005, air-conditioning energy consumption has taken an increasing proportion of total energy consumption in recent years^23^, which ensures the validity of our estimation. The reduction of HII resulting from the replacement is near 1°C. We assume the reduction of HII and air-conditioning energy saving is a linear relationship. If CVs were replaced by EVs, during the summer in Beijing, the energy consumed by air conditioners in buildings would decrease by:where *k*_2_ is the percentage of the decreased energy consumed by air conditioners in buildings.

The amount of daily energy that could be saved is 14.44 million kWh, reaching 26.75% of the total electricity consumed by EVs, as shown in equations (12) and (13):where Δ*P*_5_ is the decreased energy consumed by air conditioners in buildings with CVs replaced and *k*_3_ is the ratio of Δ*P*_5_ to energy consumed by EVs. With the decrease in air-conditioning energy consumption, less heat would be emitted, which would also contribute to mitigating UHIE and energy saving.

### Reduction of CO_2_

In 2012 in China, 740 g of CO_2_ was emitted when 1 kWh of electricity was supplied to consumers^30^. Therefore, when 14.44 million kWh are saved, CO_2_ emissions could be reduced 10,686 tonnes.

The data in this paper are mainly from the government of Beijing and the State Grid Beijing Electric Power Company. In this paper, we have to use some data of other years because some data of 2012 are not available. Therefore, our estimation of the benefits of replacing CVs with EVs is slightly lower than its actual contribution.

According to the analysis and estimation above, the replacement of CVs by EVs can substantially alleviate UHIE in the summer in metropolitan areas, which can improve the local climate, significantly reduce air-conditioning energy consumption and greenhouse gas emissions, thus helping to address global climate change.
